# Performance Analysis and Quality Evaluation of Wheat Storage in Horizontal Silo Bags

**DOI:** 10.1155/2021/1248391

**Published:** 2021-09-06

**Authors:** Mohamed M. El-Kholy, Reham M. Kamel

**Affiliations:** Agricultural Engineering Research Institute, Agricultural Research Center, Giza, Egypt

## Abstract

Wheat still suffers from the problem of traditional storage methods, limited storage capacity, and a high percentage of losses in terms of quantity and quality. Hermetic silo bags are economical and alternative technique to the traditional storage methods. Ten horizontal plastic silos with the capacity of 200 tons/silo were tested and evaluated for eight months of wheat storage. The evaluations included grain bulk temperature, CO_2_ concentration, fungal and microbial count, insect count, grain moisture content, 1000-grain weight, falling number, and protein content. The results showed that the stored wheat quality was maintained without any significant difference during the storage period in terms of 1000-grain weight, grain moisture content, and falling number, while there were slight changes in protein content and kernel hardness with a decrease of 5.5% and 4.6% at the end of the storage period. There were no statistically significant differences at the sampling location along the length of the storage silos, which confirms the homogeneity of the internal conditions of the examined silo. The grain bulk temperature inside the silos was always lower than the surrounding ambient air temperature. The higher concentration of carbon dioxide inside the silos during the storage period led to a decrease in fungal and microbial count and the presence of dead insects at the end of the storage period.

## 1. Introduction

Wheat (*Triticum aestivum vulgare* L.) is one of the most important strategic crops in the world [1]. The available storage capacity in most countries cultivating wheat is currently insecure and insufficient for crop storage capacity [2].

Documented grain postharvest loss estimates worldwide differ by grain and by region, but typically ranged from 2 to 10% [3]. The total wheat postharvest losses approached 4.32% while storage losses reached the maximum of (41.7% of the total) among all postharvest operations [4, 5]. Qualitative losses such as insect or heat damaged kernels, insect-infested, and microbial contaminated grains unfit for consumption or sale. Developing new tools to reduce the amount of grain losses after harvest, especially at the storage stage, is an important strategy to fight hunger and poverty and increase global food and nutrition security.

Various methods are used for storing wheat such as jute or burlap bags, metal silos, bulk storage in rooms, and open-air door called (Shona) [6]. During storage, the grains are exposed to serious problems in composition and quality, especially for the traditional and uncontrolled storage methods [7]. Quality losses of wheat due to improper storage method and conditions exceed 6.6% for storage in jute bags under outdoor storage conditions, and these losses can be reduced up to 2% if stored in metal silos [8]. However, the current metal silo does not provide complete protection for safe storage against insect infestation and mould. In addition, they often suffer temperature fluctuations, grains lumping due to moisture condensation on the inner wall of the silos, and also high energy consumption due to mechanical aeration process [9].

Silo bags are a relatively new form of crop storage in many countries that are used to store different types of grains such as wheat, barley, corn, soybean, sunflower, canola, and many other crops [10–14]. Storage of dry wheat grains in an economical hermetic silo bag is an alternative technique to traditional storage methods and/or metal silos. The main advantage of silo bags storage is to reduce transportation costs during harvesting season from production fields to storage sites because it can be placed in any part of the farm [15]. This technique allows the modified atmosphere storage system results from respiration of grains and microorganisms to increase CO_2_ and decrease O_2_ concentration (less than 10%). This condition reflects in reduction of biological activities of the microorganisms inside the bags and thus creates an unfavorable modified atmosphere for insects and moulds [10, 15, 16].

Previous studies of wheat storage in silo bags included mathematical modeling and determination the change in concentration CO_2_ in silo bags carrying wheat in Argentina [17]. Development of insect species is in wheat silo bags under tropical and moderate weather conditions [18]. Analysis heat and moisture transfer inside the silo bags are filled with wheat by [19]. Simulation of the change in gas concentration is as related to respiration rate and permeability of the plastic materials of bags under Argentina condition [20].

In general, this system has been successfully certified for grain storage in Argentina [18–21], Australia [22, 23], Canada [14], and other countries. However, Egyptian conditions (as representative location for the African areas in terms of weather, economic, and social conditions) differ significantly from other sites where this technology has been applied.

The main objective of the current study is to test and evaluate the hermetic storage technology of wheat in horizontal silo bags under Egyptian conditions in order to solve the problem of limited storage capacity high storage losses in quantity and quality and eliminating the need for using the nonhealthy phosphine fumigation. The effect of the wheat storage period (eight months) and sampling location on grain temperature, moisture content, CO_2_ concentration, fungal and microbial count, and insect count inside ten examined silos with capacity of 200 ton/silo were monitored. The changes in grain quality were also monthly tested and evaluated under the following methodology.

## 2. Materials and Methods

### 2.1. Preparing Grain Samples

Samples of wheat cultivar (Gimmiza-9) were harvested at a moisture content of 11.86%. w.b. The collected samples were cleaned of foreign, broken, and immature kernels before storage in the examined silo bags.

### 2.2. Methods

**Figure 1:**
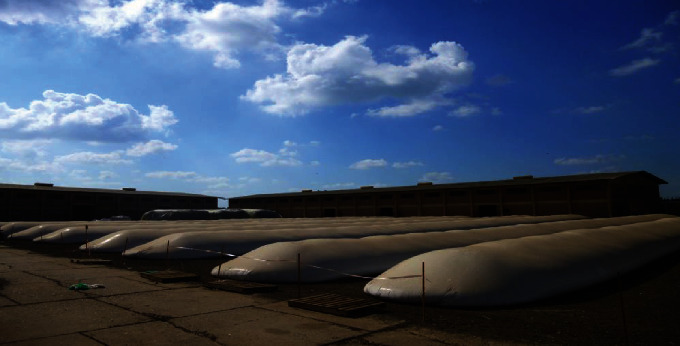
Photo for the tested horizontal silo bags (capacity 200 ton/silo).

Ten horizontal plastic silo bags with capacity of 200 tons/silo were installed in a wheat storage site at the north of Egypt (31°22^″^N–31°34^″^E) as shown in Figure 1. The silo dimensions were 2.74 m diameter and 60 m long with a polyamid film thickness of 230 micron. A white outer layer of the tested silos was assigned to reflect the ultraviolet radiation causing grain heating, and the black inner was used to prevent light transmission to the stored grain. The storage period began from June, 2020 to January, 2021. The specifications of the plastic film used for developing the tested silos are summarized in Table 1.

**Figure 2:**
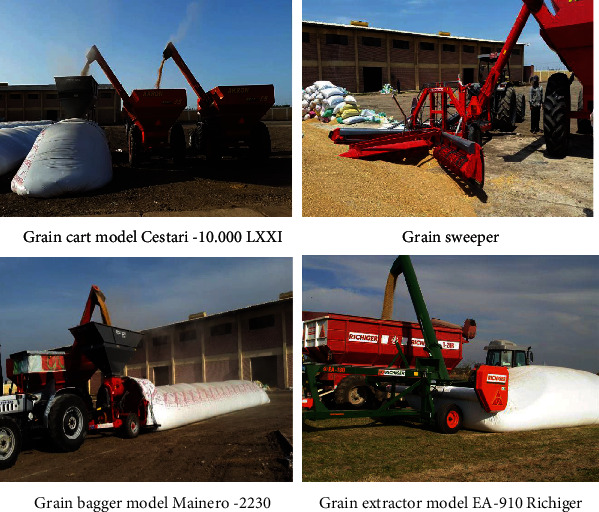
Machinery used for silo bag processing.

The grain bagger model Mainero–2230 and grain cart model Cestari-10.000 LXXI were used for charging the grain into the silo bags, and the grain extractor model EA-910 Richiger was used for grain discharging at the end of storage period (see Figure 2). For testing process, three samples were monthly collected at different locations along the length of each silos (front, middle and end of the silo). Three subsamples were also taken from each location at different depths (A-top = 0.2 m depth, B-middle = 1.37 m depth, and C-bottom = 2.54 m depth). The sampling process was conducted by penetrating the surface of plastic film using a steel probe of 1.52 m collecting 1.5 kg at each testing point/silo. The points of sampling locations were closed using a special plastic tape to keep precise sealing of silos.

### 2.3. Equipment and Measuring Procedures

#### 2.3.1. Ambient Air Temperature and Relative Humidity

A temperature meter (Model Kaye Dig. 14) with thermal sensors was used to measure the ambient air temperature at different location of storage site, and a relative humidity meter (Model Ex-Tech) was used for measuring are the relative humidity at adjacent point of temperature measurement.

#### 2.3.2. Grain Bulk Temperature

Grain bulk temperature inside the examined silos was measured at different locations of each silo using a temperature meter probe and recorded (Lutron, Model MS-7011).

#### 2.3.3. Grain Moisture Content

The wheat moisture content was determined using the standard method [24]. 10 grams of wheat grain was placed in an electric air oven at 130°C for 16 h, and then they were kept in a desecrator under room temperature for 15 min and weighted by a digital balance with accuracy 0.001 g.

#### 2.3.4. 1000-Grain Weight

1000 grains were counted and then weighted using a precision electronic scale (accuracy 0.001 g).

#### 2.3.5. Carbon Dioxide Concentration

CO_2_ concentration was monitored monthly at different locations of each silo by O_2_ and CO_2_ Analyzer (VIGAS, Model Box-121).

#### 2.3.6. Fungal and Microbial Count

Grain samples were monthly collected from different locations of each silo to determine mold prevalence (cfu/g of grains), using the procedure described by [25]. 25 g of representative samples were soaked in 250 ml of sterile peptone (0.1%) water for 30 min before digestion for 2 min. 1 ml of the sample, serially diluted in 9 ml of peptone water and a 100 *μ*l sample from the serial dilution, was drop plated on dichloron glycerol-18 (DG-18) agar medium (Oxoid chemicals, Hampshire, UK) and incubated at 35°C for 4-5 days. After incubation, the former colonies were recorded (cfu/g).

#### 2.3.7. Insect Count

Grain samples from different locations of the tested silos were sieved, and the insect pests were identified according to [24].

#### 2.3.8. Protein Content

The percentage of the crude protein was measured as N concentration and converted to protein by multiplying this percentage by the nitrogen constant factor. The standard semimicro kijeldahl method was used according to [14, 22, 26].

#### 2.3.9. Falling Number

Falling number of the flour obtained from stored wheat was determined according to the [17] method. A suspension of flour was prepared by adding 25 ml of distilled water to 7 g of wheat flour (14% d.b) in two falling number tubes. The suspension was heated to gelatinize the starch of flour, and time counted in secs to drop down a plunger having definite weight into the gelatinized flour paste was recorded as falling number.

#### 2.3.10. Kernel Hardness

The hardness meter (Model SHIMPO, FGC-50) was used for determination of kernel hardness [27].

### 2.4. Statistical Analyses

Measuring values of different tested factors were represented as the mean ± SD (standard deviation) for ten replicates. A one-way or two-way analysis of variance (ANOVA) and the correlation coefficient [28] were conducted by the SPSS 19.0 software, and the least significant difference (LSD) was determined by using multiple range tests. *p* values less than 0.05 were considered significant.

## 3. Results and Discussion

### 3.1. Ambient Weather Condition of Storage Site

The change in weather condition surrounding the silos during the storage period is presented in Table 2. The average ambient air temperature ranged from 17.6 to 29.9°C, while the average relative humidity ranged from 59.9 to 70.1%, and rainfall was about 2.86 from Nov., 2020 to Jan., 2021.

### 3.2. Grain Bulk Temperature

The change in grain mean bulk temperature usually follows the same trend of the change in ambient air temperature, where the average bulk temperature at the beginning of the storage period was 26.65°C (June, 2020) and decreased to 12.43°C (Jan., 2021) at the end of storage period (see Table 3). According to ANOVA analysis, the grain bulk temperature was significantly different among months (*p* < 0.05; LSD0.05 = 0.30). Meanwhile, the grain bulk temperature was lower than the daytime ambient temperature due to the effect of white surface layer of silos (Anti-UV), which reflects most of the ultraviolet rays that causing the stored grains to overheat as reported by [29] who mentioned that the outside surface of the metal silo is painted white to reduce the temperature rise of the grains. The results confirm that the temperature of the grains inside the silo is below the permissible limit for insect development and growth. Furthermore, [21, 23] revealed that the optimum temperature for biological activities of storage insects ranges from 26 to 33°C and when ambient temperatures are outside this range, these activities are reduced. The lower grain temperature could also be attributed to the lower grain respiratory rate, thus avoiding early spoilage during storage period as mentioned by [30] who found that the respiration rate of wheat grain increased after several days when grain temperature increased to 30 and 35°C.

### 3.3. Grain Moisture Content

Table 4 shows a slight increase in moisture content of grains ranged from 0.27 to 1.91% which indicating no reabsorption of moisture from the ambient atmosphere due to the water sealing effect of the plastic film which is characterized by its ability to prevent stored grains from condensed moisture on the surface of silos as well as the rains during winter. These results agreed with the studies conducted by [6, 9, 16]. In general, the storage period, sample location, and interaction between them showed no significant difference by a two-way ANOVA (*p* > 0.05). The results of stability stored in the wheat moisture content also indicate the absence of effective fungal or microbial growth that leads to an increase in grain moisture content [31].

### 3.4. 1000-Grain Weight

No losses in the grain weight were detected which reflects the absence of any insect growth as shown in Table 4. Analysis of variance indicated no significant differences for storage period, sampling location, and interaction between them (*p* > 0.05) on 1000-grain weight by two-way ANOVA. These results are in agreement with [32] who stated that moisture content played a significant role in weight loss of grains.

### 3.5. Carbon Dioxide Concentration

Carbon dioxide concentration also showed significant differences along the storage period (df = 7; *p* < 0.05; LSD_0.05_ = 0.32) but the differences was not significant for the sampling location and the interaction between the sampling location and the storage time (df = 2, 14; *p* > 0.05). From Table 5, it was clear that the horizontal plastic silos could control the concentration of carbon dioxide gas within the stored grain mass at the safe level for microbes and insect inhibition, meaning that it represents one of the ecosystems as a typical modified atmosphere storage.

The results showed that carbon dioxide levels ranged from 5.39 to 7.27% during the storage period. In comparison with [20], carbon dioxide remained above 12% for stored wheat (12-13 w.b) after six months, while it reached 4.6% in [26] with a difference of 3% between the summer and winter months. Therefore, it should be noted that the difference in carbon dioxide percentages inside the silos depends on the respiration rate of grains and the level of microorganisms inside the silos in addition to permeability of the plastic film and its ability to maintain the appropriate gaseous balance for the storage process [16, 32, 33].

### 3.6. Fungal and Microbial Count

**Figure 3:**
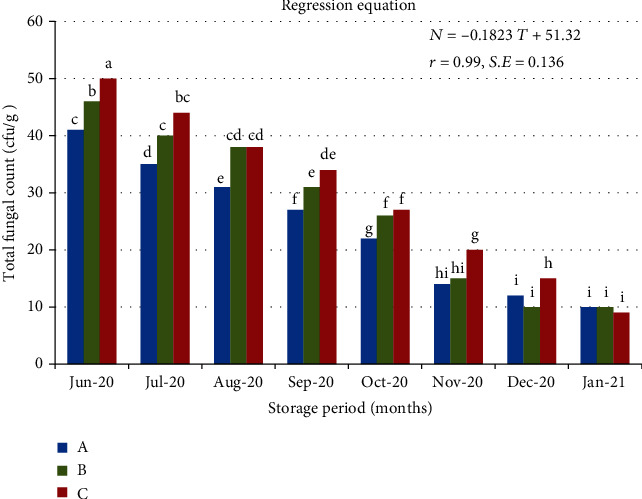
Changes in total fungal count inside the examined silos (cfu/g) as related to the storage period at different sampling locations A, B, and C.

As shown in Figure 3, the average fungal count inside the ten examined silos at the beginning of storage period ranged from 41 to 50 cfu/g, while the average fungal count decreased to a range of 9 to 10 cfu/g at the end of storage period. This means that the fungal count decreased with the increase of storage period, as this is due to the high percentage of carbon dioxide, especially during the months of November and December. This result is similar to [34] which showed that the fungal growth of stored durum wheat significantly decreased after 5-6 months of storage under 2-5% CO_2_ concentration in the silo bag (located in north Italy). Total fungal account also is low compared to [28, 34]; so, the inability of the existing fungi to produce mycotoxins during the storage period was clean. Fungal counts varied for the storage period (df = 7; *p* < 0.05; LSD_0.05_ = 5.73) and for sampling location within the bags (df = 2; *p* < 0.05; LSD_0.05_ = 3.51), but the interaction of the storage period and sampling location was not significant (*p* > 0.05) by two-way analysis of variance ANOVA, as mentioned by [28, 33].

**Figure 4:**
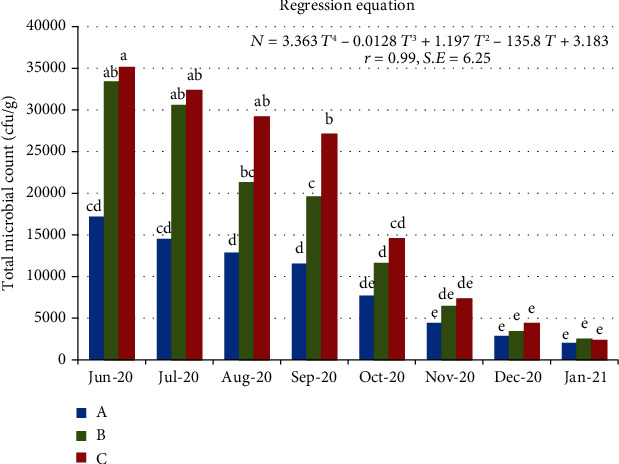
Changes in total microbial count inside the storage silos (cfu/g) as related to the storage period at different sampling locations A, B, and C.

Figure 4 presents the change in total microbial count/g of grains as related to storage time. The microbial count ranged from 17190 to 35100 cfu/g at the beginning of storage period, while the average total microbial count decreased with increasing the storage period and reached the lowest values at the end of the storage period (2022 to 2502 cfu/g). The results clarified that total microbial counts varied with the storage period (df = 7; *p* < 0.05; LSD_0.05_ = 10727.92) and by sampling location within the bags (df = 2; *p* < 0.05; LSD_0.05_ = 6569.48), but the interaction of storage period and sampling location was not significant (*p* > 0.05) as indicated from the two-way ANOVA analysis.

In general, as explained by [29, 35], both the number of fungi and total microbial count are affected by the initial state of grains, the percentage of carbon dioxide inside the silo, and the moisture content of grains.

### 3.7. Insect Count

Insect growth is one of the most important factors affecting the safe storage period and grain quality. Some species of insects such as *Oryzaephilusfu surinamesis* and *Rhyzopertha dominica* are able to develop by a percentage of 35% at low humidity [36]. These insects make holes in the seed and feed on it, as well as help in providing a good environment for fungal and microbial growth as explained by [10].

As shown in Table 6, some insect growths began to appear during the storage month (August) as a result of high ambient temperature and relative humidity with the average count ranged from 0.2 to 0.4 insects/kg, but most of these insects were not survive due to the high percentage of carbon dioxide inside the silos and the low grain moisture content. The effect of the storage period was significant regarding insect count (df = 7; *p* < 0.05; LSD_0.05_ = 0.23) especially between summer and winter seasons. However, sampling location and interaction between the storage period and sampling location were not significant (*p* > 0.05). The obtained results agree with [37], and they confirmed that insect numbers and seed spoilage of wheat were completely controlled at a concentration of 20% carbon dioxide within two months of the modified atmosphere storage period, and concentrations of 5 and 10% of carbon dioxide also led to a significant reduction in insect growth and seed spoilage within 6 months compared to ambient condition.

Viable insects did not affect the grains due to their weak activity and their inability to penetrate the surface of the grain, especially the weevil insect [20, 17]. In general, the stored grains clearly showed no insect infestation during the entire storage period.

### 3.8. Protein Content and Falling Number

As shown in Table 7, the average percentage of the protein content during the first month of storage was 11.10% d.b, while it was 10.49% d.b. at the end of storage period. This means that storage in silo bags led to a slight decrease in protein content, equivalent to 5.5%. One-way ANOVA analysis of variance confirmed that the protein content was significantly different along the storage period (df = 7; *p* < 0.05; LSD_0.05_ = 0.31). [38] reported that there was a significant decrease in the protein content for stored wheat in silo bags which decreased from 12.6% to 10.8% during the storage period.

The average falling number for the stored wheat ranged from 427.60 s at the first month of storage to 438.30 s at the end of storage period, which means that it was not affected by the storage period, as illustrated in Table 7. Falling number was not significant (*p* > 0.05) along the storage months. The falling number was higher than 250 s which means that the enzyme activity in the tested grain or flour samples is ideal for bread production. The falling number less than 250 s means increased activity of the amylase hydrolyzate enzyme, which leads to producing flour that is not suitable for bread making [39]. In general, the falling number remained in a good range (250-350) with reference to baking quality [40].

### 3.9. Kernel Hardness

The average of kernel hardness during the first month of storage ranged from 48.80 to 48.88 N, while it was slightly decreased between 51.04 and 51.17 N with a decrease equivalent to 4.6% at the end of storage period as shown in Table 8. From the abovementioned results, the kernel hardness decreased slightly due to the slight increase in the moisture content of grains which causes a decrease in their hardness. Wetting effect of wheat grains on their hardness was studied by [41], and the results showed a significant decrease in grain hardness. The mean difference of hardness differed significantly with the storage period (df = 7; *p* < 0.05; LSD_0.05_ = 1.71) but no significant observed with the sampling location (*p* > 0.05) by two-way ANOVA.

## 4. Conclusion

Storage of freshly harvested wheat grain at the initial moisture content of 11.86% w.b in horizontal silo bags with capacity of 200 ton/silo for 8 months showed a good quality results of stored grain in terms of relatively stable moisture content and grain weight, lower fungal and microbial count, and minimum insect infestation. A slight reduction in the protein content and kernel hardness was detected while the falling number of the wheat flour was suitable for high baking quality. In general, the silo bag storage method is recommended for application in wheat farms as a solution for limited safe storage capacity with minimum quality and quantity losses.

## Figures and Tables

**Table 1:** Specifications of the tested plastic silo film.

Property	Unit	Method	Value		
			Max.	Min.	Mean
Average thickness	*μ*m	DIN 53370	251	243	250
2 SEGMA thickness tolerance	%		5.6	3.8	4.7
Width	mm	Internal	442	442	442
Coefficient of friction					
Out/out	—	ASTM D 1894	0.40	0.36	0.38
In/in			0.20	0.18	0.19
NTR/M		ISO 8295	—	—	—
Surface tension	Dyn/CM	DNI ISO 8296	—	38	—
Tensile strength at break MD	Mpa	ASTM D882	50.2	42.2	46.6
Tensile strength at break TD	Mpa		46.1	41.2	43.4
Tensile strength at yield MD	Mpa		19.1	15	17.3
Tensile strength at yield TD	Mpa		21.8	20.4	21
Elongation at break MD	%		569.4	475.7	531.7
Elongation at break TD	%		591.6	524.5	563.2
Elongation at yield MD	%		7.8	6.7	7.3
Elongation at yield TD	%		8.1	7.1	7.8
Oxygen permeability	Cc/m^2^/day				≤450
Water vapor permeability	g/m^2^/day				≤2

**Table 2:** Ambient air temperature (°C), relative humidity (%), and rainfall (mm) during the storage period (June, 2020-Jan, 2021).

Storage period	Ambient temperature, °C			Air relative humidity, %			Rainfall, mm		
	Mean ± SD (°C)	Maximum	Minimum	Mean ± SD (%)	Maximum	Minimum	Mean ± SD (%)	Maximum	Minimum
June, 2020	24.87 ± 5.85	31.60	21.00	60.50 ± 20.60	81.40	40.20	0.00 ± 0.00	0.00	0.00
July, 2020	29.17 ± 4.84	33.60	24.00	66.00 ± 17.55	82.70	47.70	0.00 ± 0.00	0.00	0.00
Aug., 2020	28.20 ± 4.75	32.50	23.10	68.97 ± 14.73	83.10	53.70	0.00 ± 0.00	0.00	0.00
Sept., 2020	27.30 ± 4.95	31.80	22.00	64.43 ± 15.63	79.40	48.20	0.00 ± 0.00	0.00	0.00
Oct., 2020	22.50 ± 4.65	26.50	17.40	59.40 ± 15.31	74.30	43.70	0.00 ± 0.00	0.00	0.00
Nov., 2020	21.63 ± 4.14	25.40	17.20	66.30 ± 14.05	78.80	51.10	1.38 ± 0.81	2.79	0.00
Dec., 2020	21.03 ± 4.44	25.10	16.30	65.93 ± 12.23	77.60	53.20	2.88 ± 0.53	3.30	2.28
Jan., 2021	17.23 ± 4.26	21.30	12.80	63.04 ± 15.23	79.30	49.10	4.32 ± 1.16	5.33	3.06

Results are expressed as means ± SD (*n* = 7) at 0.05 probability level (one-way ANOVA).

**Table 3:** Change in bulk temperature (°C) as related to storage time with regression equation.

Storage period	Mean ± SD (°C)	Maximum	Minimum
June, 2020	25.65 ± 0.40a	26.33	25.13
July, 2020	24.51 ± 0.18b	24.73	24.27
Aug., 2020	24.62 ± 0.19b	24.93	24.33
Sept., 2020	22.99 ± 0.13c	23.17	22.70
Oct., 2020	17.57 ± 0.00d	17.57	17.57
Nov., 2020	15.76 ± 0.41e	16.53	15.30
Dec., 2020	14.10 ± 0.43f	14.51	13.07
Jan., 2021	12.43 ± 0.55g	13.22	11.75
Regression equation	*N* = −1.122*T*^5^ + 7.67 *T*^4^ − 0.00019 *T*^3^ + 0.0205 *T*^2^ − 0.9711 *T* + 40.84		
	*r* = 0.99	S.E = 0.08	

^∗^The same lowercase letters mean no significant difference at 0.05 probability level (one-way ANOVA). Results are expressed as means ± SD (*n* = 7). ^∗^*r* and *S*.*E* are correlation coefficient and standard error estimation of bulk temperature changes, respectively, in wheat during storage time.

**Table 4:** Change in grain moisture content (% w.b) and 1000-grain weight (g) as related to the storage period and sampling location.

Storage period, month	Sampling location					
	Grain moisture content, % (w.b.)			1000-grain weight, g		
	A	B	C	A	B	C
June-2020	11.86 ± 0.43	11.91 ± 0.43	11.90 ± 0.43	40.51 ± 0.93	40.65 ± 0.94	40.66 ± 0.91
July-2020	11.83 ± 0.41	11.89 ± 0.43	11.86 ± 0.42	40.39 ± 0.79	40.64 ± 0.93	40.52 ± 0.79
Aug.-2020	11.85 ± 0.42	11.86 ± 0.44	11.90 ± 0.43	40.50 ± 0.94	40.65 ± 0.92	40.67 ± 0.91
Sep.-2020	11.96 ± 0.46	11.88 ± 0.42	11.88 ± 0.41	40.48 ± 0.85	40.65 ± 0.93	40.64 ± 0.89
Oct.-2020	11.96 ± 0.47	11.89 ± 0.42	11.89 ± 0.43	40.51 ± 0.92	40.65 ± 0.96	40.65 ± 0.89
Nov.-2020	11.87 ± 0.43	11.92 ± 0.42	11.90 ± 0.43	40.54 ± 0.94	40.67 ± 0.96	40.66 ± 0.89
Dec.-2020	12.02 ± 0.43	12.02 ± 0.42	12.00 ± 0.44	40.80 ± 0.89	40.91 ± 0.93	40.81 ± 0.89
Jan.-2021	12.08 ± 0.43	12.13 ± 0.44	12.09 ± 0.44	40.92 ± 0.92	41.00 ± 0.94	40.93 ± 0.92

Results are expressed as means ± SD (*n* = 14) at 0.05 probability level (two-way ANOVA).

**Table 5:** Change in CO_2_ concentration (%) inside the tested silos as related to storage period and sampling location.

Storage period, month	Sampling location		
	A	B	C
June-2020	5.64 ± 0.76e	5.39 ± 0.79e	5.63 ± 0.45e
July-2020	5.75 ± 0.74de	5.73 ± 0.60de	5.83 ± 0.43de
Aug.-2020	5.97 ± 0.81d	5.96 ± 0.72de	6.11 ± 0.49cd
Sep.-2020	6.39 ± 0.56c	6.37 ± 0.68c	6.49 ± 0.62bc
Oct.-2020	7.01 ± 0.82ab	7.27 ± 0.51a	7.05 ± 0.57ab
Nov.-2020	6.76 ± 0.89b	7.08 ± 0.51ab	6.71 ± 0.76bc
Dec.-2020	5.94 ± 0.59de	6.00 ± 0.64d	5.97 ± 0.60d
Jan.-2021	2.09 ± 0.39f	1.92 ± 0.45f	1.94 ± 0.50f
Regression equation	*N* = −1.955 *T*^4^ + 7.381*T*^3^ − 0.0009*T*^2^ + 0.0508 *T* + 4.646		
	*r* = 0.99	S.E = 0.08	

^∗^The same lowercase letters mean no significant difference at 0.05 probability level (two-way ANOVA). Results are expressed as means ± SD (*n* = 14). ^∗^*r* and *S*.*E* are correlation coefficient and standard error estimation of CO_2_ changes, respectively, in wheat during storage time.

**Table 6:** Insect count inside the tested silos (insect/kg).

Storage period, month	Sampling location		
	A	B	C
June-2020	0.00 ± 0.00c	0.00 ± 0.00c	0.00 ± 0.00c
July-2020	0.00 ± 0.00c	0.00 ± 0.00c	0.00 ± 0.00c
Aug.-2020	0.20 ± 0.04bc (viable)	0.40 ± 0.07b (viable)	0.20 ± 0.04bc (viable)
Sep.-2020	0.00 ± 0.00c	0.20 ± 0.04bc (dead)	0.00 ± 0.00c (dead)
Oct.-2020	0.30 ± 0.05b (dead)	0.20 ± 0.04bc (dead)	0.00 ± 0.00c (dead)
Nov.-2020	0.20 ± 0.04bc	0.30 ± 0.05b	0.30 ± 0.07b
Dec.-2020	0.20 ± 0.06bc (dead)	0.04 ± 0.07b (dead)	0.40 ± 0.05b (dead)
Jan.-2021	0.00 ± 0.00c	0.70 ± 0.09a (dead)	0.70 ± 0.08a (dead)
Regression equation	*N* = −3.429 *T*^7^ + 3.443 *T*^6^ − 1.423*T*^5^ + 3.109 *T*^4^ − 0.00038 *T*^3^ + 0.0261 *T*^2^ − 0.8902 *T* + 11.33		
	*r* = 0.99	S.E = 0.004	

^∗^The same lowercase letters mean no significant difference at 0.05 probability level (two-way ANOVA). Results are expressed as means ± SD (*n* = 14). ^∗^*r* and *S*.*E* are correlation coefficient and standard error estimation of insect count changes, respectively, in wheat during storage time.

**Table 7:** Protein content inside silos (% d.b) and falling number (s) as related to storage period.

Storage period, month	Protein content, % d.b			Falling number, s		
	Mean ± SD	Maximum	Minimum	Mean ± SD	Maximum	Minimum
June-2020	11.10 ± 0.26 a	10.66	11.60	427.60 ± 25.05	385.00	464.00
July-2020	10.85 ± 0.36 ab	10.30	11.35	429.60 ± 20.86	395.00	460.00
Aug.-2020	10.63 ± 0.39 b	10.00	11.20	403.90 ± 43.00	320.00	455.00
Sep.-2020	10.65 ± 0.37 b	10.24	11.40	411.80 ± 50.80	330.00	464.00
Oct.-2020	10.64 ± 0.37 b	10.25	11.51	423.00 ± 26.69	380.00	470.00
Nov.-2020	10.73 ± 0.31 b	10.19	11.31	445.30 ± 36.28	398.00	490.00
Dec.-2020	10.55 ± 0.40 b	10.00	11.00	435.10 ± 49.70	339.00	486.00
Jan.-2021	10.49 ± 0.38 b	9.73	11.17	438.30 ± 28.97	396.00	495.00
Regression equation	*N* = 3.62 *T*^6^ − 2.381 *T*^5^ + 5.632 *T*^4^ − 5.944 *T*^3^ + 0.00033 *T*^2^ − 0.0185 *T* + 11.48					
	*r* = 0.98	S.E = 0.004				

^∗^The same lowercase letters mean no significant difference at 0.05 probability level (one-way ANOVA). Results are expressed as means ± SD (*n* = 7). ^∗^*r* and *S*.*E* are correlation coefficient and standard error estimation of protein changes, respectively, in wheat during storage time.

**Table 8:** Changes in kernel hardness (*N*) inside the silos during the storage period and sampling locations A, B, and C.

Storage period, month	Sampling location		
	A	B	C
June-2020	51.25 ± 2.04ab	51.04 ± 1.94ab	51.17 ± 1.97ab
July-2020	51.11 ± 2.05ab	51.39 ± 1.66a	51.40 ± 1.85a
Aug.-2020	50.25 ± 3.84ab	50.20 ± 3.80ab	50.30 ± 3.92ab
Sep.-2020	49.35 ± 3.57b	49.35 ± 3.93b	49.55 ± 3.61b
Oct.-2020	48.55 ± 4.19b	49.35 ± 3.93b	49.25 ± 3.87b
Nov.-2020	49.25 ± 3.98b	49.20 ± 3.82b	49.25 ± 3.87b
Dec.-2020	49.00 ± 3.85b	49.03 ± 3.77b	48.97 ± 3.70b
Jan.-2021	48.88 ± 3.88b	48.66 ± 1.76b	48.80 ± 3.66b
Regression equation	*N* = −1.941 *T*^4^ + 1.018 *T*^3^ − 0.0017 *T*^2^ + 0.1134 *T* + 46.62		
	*r* = 0.98	S.E = 0.098	

^∗^The same lowercase letters mean no significant difference at 0.05 probability level. Results are expressed as means ± SD (*n* = 14). ^∗^*r* and *S*.*E* are correlation coefficient and standard error estimation of kernel hardness changes, respectively, in wheat during storage time.

## Data Availability

The data used to support the findings of this study are included within the article.
